# Supplementary material for: Lithium Chloride Sensitivity in Yeast and Regulation of Translation
Source: Int J Mol Sci. 2020 Aug 10;21(16):5730. doi: 10.3390/ijms21165730 (PMC7461102; doi:10.3390/ijms21165730)
Supplement: Supplementary file 1 [file ijms-21-05730-s001.pdf]

Supplementary information

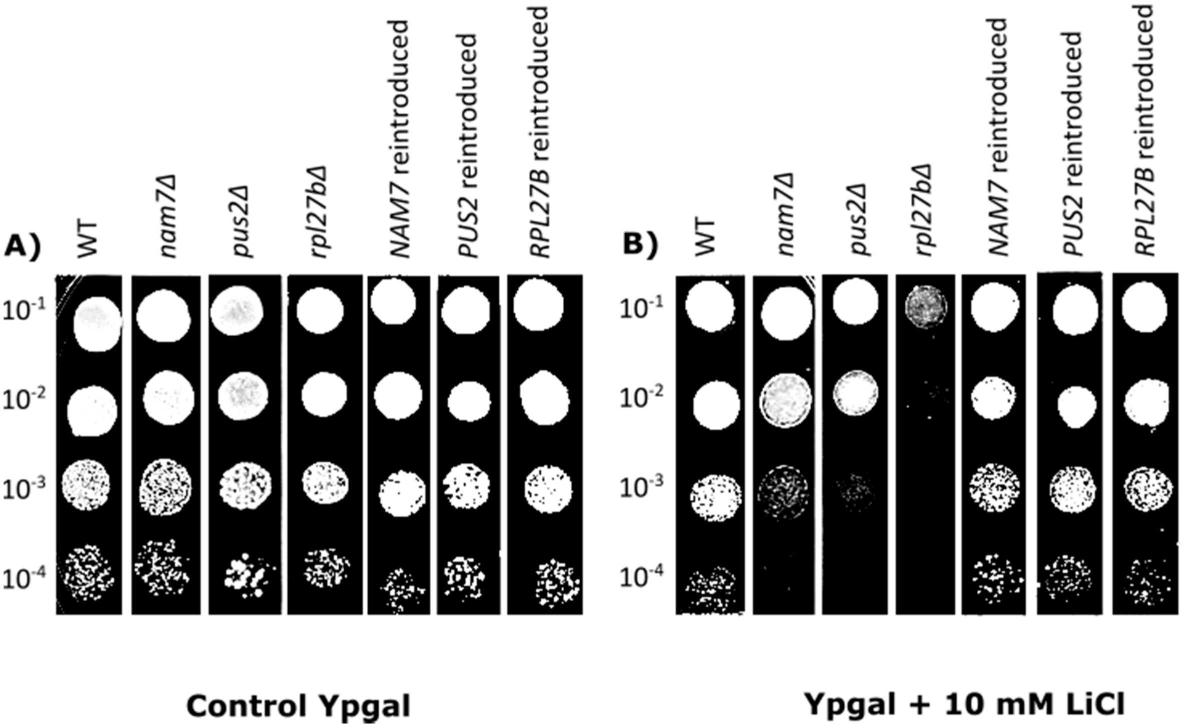

Figure S1. LiCl sensitivity analysis for different yeast strains using spot test analysis. Yeast cells were serially diluted as indicated (10^{-1} to 10^{-4}) and spotted on YPgal media with or without LiCl (10 mM). Growth sensitivity of *nam7Δ*, *pus2Δ*, and *rpl27bΔ* are compared to strains where deleted genes are reintroduced back into the genome, on media without (**A**) or with LiCl (10 mM) (**B**). Reintroduction of the deleted genes back into the chromosome of the corresponding gene deletion mutants reversed the observed growth reduction.

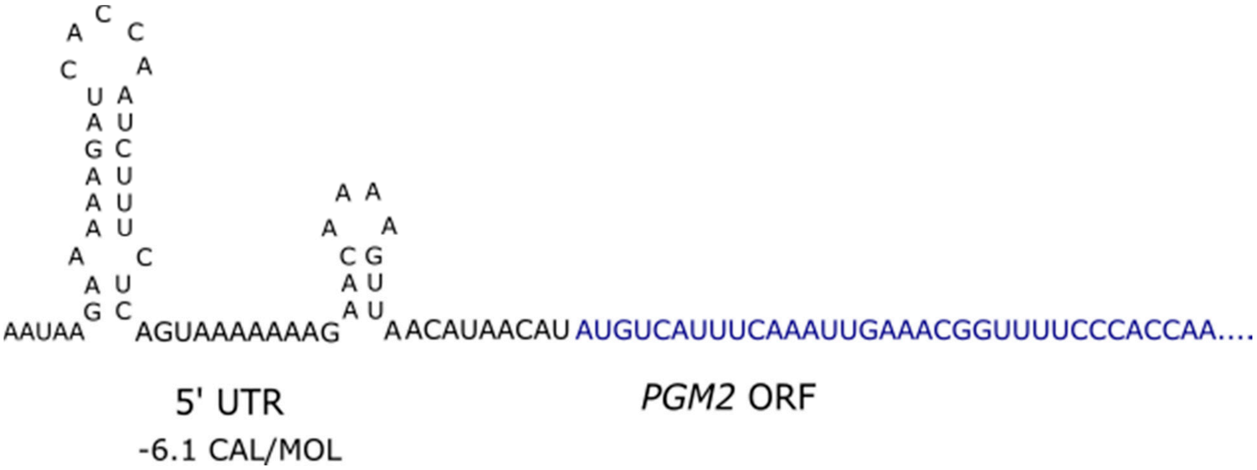

Figure S2. The secondary structure of PGM2 5′ UTR. Unlike most yeast ORFs, the 5′ UTR of PGM2 is thought to be structured (Tuller et al., 2009).
